# Reporting errors in plain radiographs for lower limb trauma—a systematic review and meta-analysis

**DOI:** 10.1007/s00256-021-03821-9

**Published:** 2021-06-18

**Authors:** Thomas York, Christopher Franklin, Kate Reynolds, Greg Munro, Heloise Jenney, William Harland, Darren Leong

**Affiliations:** 1 Guys and St Thomas’ NHS Trust, St Thomas’ Hospital, London, UK; 2 London Northwest University Healthcare NHS Trust, London, UK

**Keywords:** Trauma radiographs, Skeletal radiographs, Lower limb, Foot and ankle, Knee, Emergency, Initial reporting, Accuracy, Reporting errors

## Abstract

**Introduction:**

Plain radiographs are a globally ubiquitous means of investigation for injuries to the musculoskeletal system. Despite this, initial interpretation remains a challenge and inaccuracies give rise to adverse sequelae for patients and healthcare providers alike. This study sought to address the limited, existing meta-analytic research on the initial reporting of radiographs for skeletal trauma, with specific regard to diagnostic accuracy of the most commonly injured region of the appendicular skeleton, the lower limb.

**Method:**

A prospectively registered, systematic review and meta-analysis was performed using published research from the major clinical-science databases. Studies identified as appropriate for inclusion underwent methodological quality and risk of bias analysis. Meta-analysis was then performed to establish summary rates for specificity and sensitivity of diagnostic accuracy, including covariates by anatomical site, using HSROC and bivariate models.

**Results:**

A total of 3887 articles were screened, with 10 identified as suitable for analysis based on the eligibility criteria. Sensitivity and specificity across the studies were 93.5% and 89.7% respectively. Compared with other anatomical subdivisions, interpretation of ankle radiographs yielded the highest sensitivity and specificity, with values of 98.1% and 94.6% respectively, and a diagnostic odds ratio of 929.97.

**Conclusion:**

Interpretation of lower limb skeletal radiographs operates at a reasonably high degree of sensitivity and specificity. However, one in twenty true positives is missed on initial radiographic interpretation and safety netting systems need to be established to address this. Virtual fracture clinic reviews and teleradiology services in conjunction with novel technology will likely be crucial in these circumstances.

## Introduction

In February of 1896, at the physics laboratory of Dartmouth College, Edwin Brant Frost used what were then known as roentgen rays to capture an image of the healing ulna of his patient, Edward McCarthy [1]. The supreme clinical applications of this novel technology were not lost on early observers, Silvanus P. Thompson (President of the Roentgen Society) said a year later:

‘Excepting only the introduction into surgery by Lord Lister of antiseptics, and the discovery of anaesthetics, no discovery in the present century has done so much for operative surgery as this of the roentgen rays’ [1].Over the following 130 years of clinical practice, plain radiographs have remained foundational to the investigation of musculoskeletal injuries. The WHO estimates that 3.6 billion investigations using ionising radiation are performed globally each year, the majority of which being simple X-rays [2]. In the UK, more than 60% of emergency department attendances have a primary diagnosis relating to the musculoskeletal (MSK) system [3]. In total, 38.7% of all patients will receive at least one plain radiograph and in MSK injuries this rises to over 50% [4].

Despite being ubiquitous, the interpretation of skeletal radiographs is challenging, and errors can be of significant detriment to both patients and care providers. The interpretation of radiographs in a trauma setting is especially fraught, with high patient turnover and often junior staff. Consequently, emergency departments are recognised as ‘high risk’ for diagnostic error [5]. Research reviewing UK medicolegal claims in skeletal radiology between 1995 and 2006, showed the ‘great majority followed missed diagnoses of fractures following trauma’ [6].

Existing research has shown variable levels of performance in the initial interpretation of skeletal radiographs for trauma. Across all radiographs in the emergency department setting, an error rate of approximately 3% has been shown [7]. In the upper limb, estimates suggest incorrect assessment is made in around 8.5% of cases [7, 8].

There have so far been limited attempts to produce summary rates of reporting error in plain skeletal radiographs of lower limb trauma, despite a body of individual studies assessing this both in generality and by more specific anatomical site.

The aims of this study were to conduct a systematic review and meta-analysis of the existing literature to establish sensitivity, specificity, and diagnostic odds ratio for the initial interpretation of lower limb radiographs (including those of anatomical sub-divisions; foot, ankle, knee and femur).

## Methods

### Review protocol and search strategy

This systematic review was prospectively registered with the PROSPERO database, a copy of the review protocol can be found under registration number CRD42020197973.

**Fig. 1:**
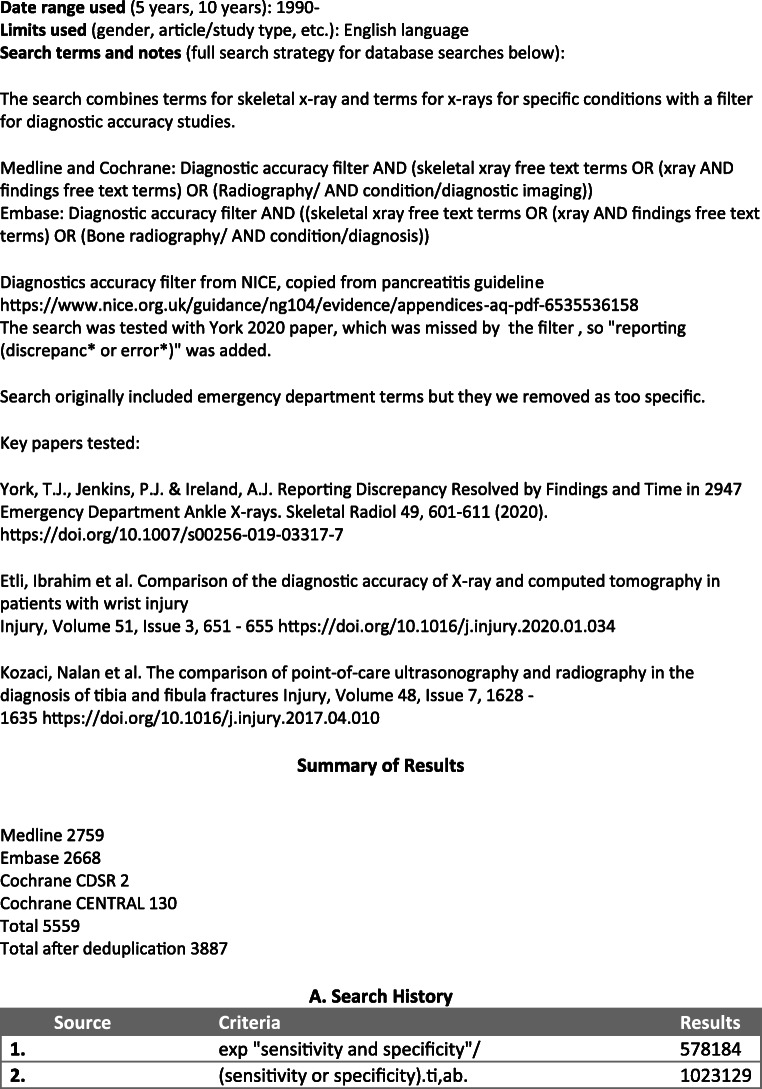

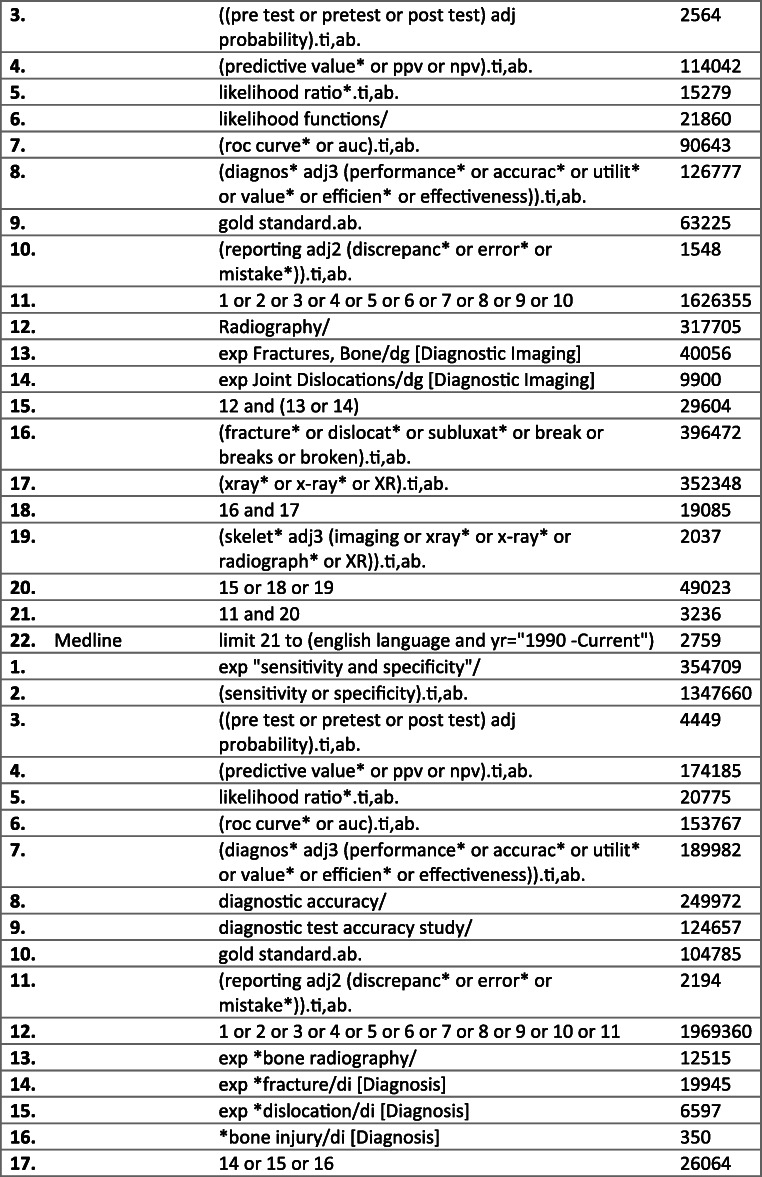

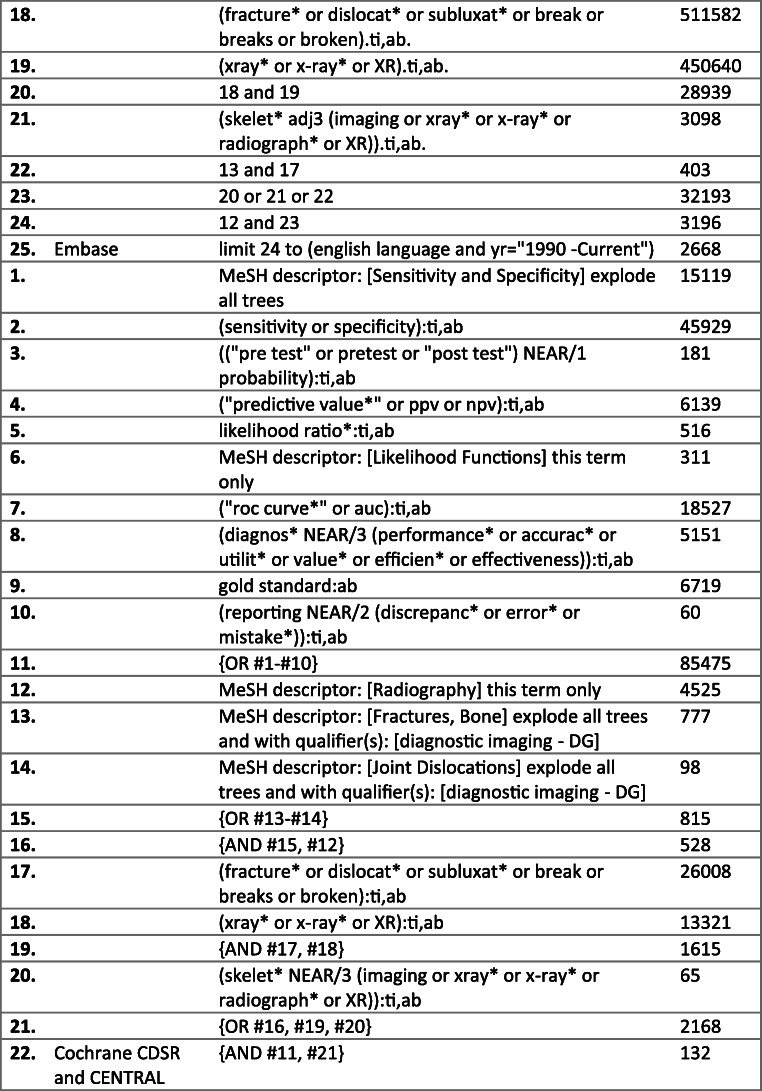
Literature review process

In April of 2020, the PubMed MEDLINE, Embase, Cochrane Database of Systematic Reviews (CDSR) and Cochrane Central Register of Controlled Trials (CENTRAL) databases were scrutinised from 1990 to the present, using a search strategy developed with the aid of Imperial College Library Services. The full electronic search strategy is detailed in Fig. 1.

### Eligibility criteria

In accordance with the objectives of this study, eligibility criteria were developed by the authors to identify papers containing pertinent data for inclusion. These were as follows:

- Written in the English language
- Conducted during or after 1990
- Original research, published in peer reviewed, academic journals (editorial letters, opinion pieces and expert reviews were excluded)
- Reporting an initial assessment of plain radiographs of the lower limb, performed by identified members of staff or grade of staff and compared to a definitive assessment of findings
- Investigation of subjects with a confirmed or suspected trauma and orthopaedic injury, as characterised by the WHO ICD 11
- Radiographs included for review being of skeletally mature subjects
- Conducted in active healthcare settings where diagnostic services are provided to a patient population
- Outcomes reported with respect to accuracy, specificity or sensitivity of radiograph reporting
- Outcomes reported with respect to specific anatomical site or regional anatomy

### Study selection

An initial sample of 200 search results was reviewed for inclusion by the six reviewing authors (TY, CF, KR, GM, HJ, WH). Using the eligibility criteria against title and abstract, each author sorted these 200 results into ‘reject’ or ‘further review’ categories. Inter-reader reliability assessment was then performed to establish the degree of agreement amongst the authors on those articles meriting further review. Fleiss’ Multirater Kappa was calculated to be 0.640 (*p* < .005), conventionally taken to represent substantial agreement [9].

Each author then individually assessed an equal share of the remaining results by title and abstract, again categorising as ‘reject’ or ‘further review’. These, along with the reviewed results of the initial sample were combined, and further categorised on the basis of the anatomical region to which they related: lower limb, upper limb, pelvis, spine and thorax, skull and facial. Where an article included data pertinent to more than one anatomical region, it was duplicated, and a copy assigned to both.

TY and CF then reviewed the full text of all potentially eligible results categorised as lower limb against the aforementioned eligibility criteria. Where disparity arose, it was resolved by means of further review and joint assessment.

### Data collection and assessment

A bespoke data extraction tool was developed by the authors; this was applied to all included studies. Variables recorded were radiograph reporting population, male/female % of radiograph subjects, recruitment methods to study, anatomical site identified, reporting accuracy/error rate %, specificity %, sensitivity % and qualitative outcome statement.

An assessment was made of methodological quality using the MINORS tool [10] and of risk of bias using a modified Cochrane RoB2 tool [11]. Where the authors initially made a divergent assessment of any study, a consensus evaluation was formed.

### Summary and synthesis

The radiograph reporting populations, reporting accuracy and specificity/sensitivity were identified as the principle summary measures. Meta-analysis was then performed in order to produce summary estimates of specificity and sensitivity, including covariates by anatomical site, using HSROC and bivariate model analysis.

## Results

### Study selection and characteristics

**Fig. 2:**
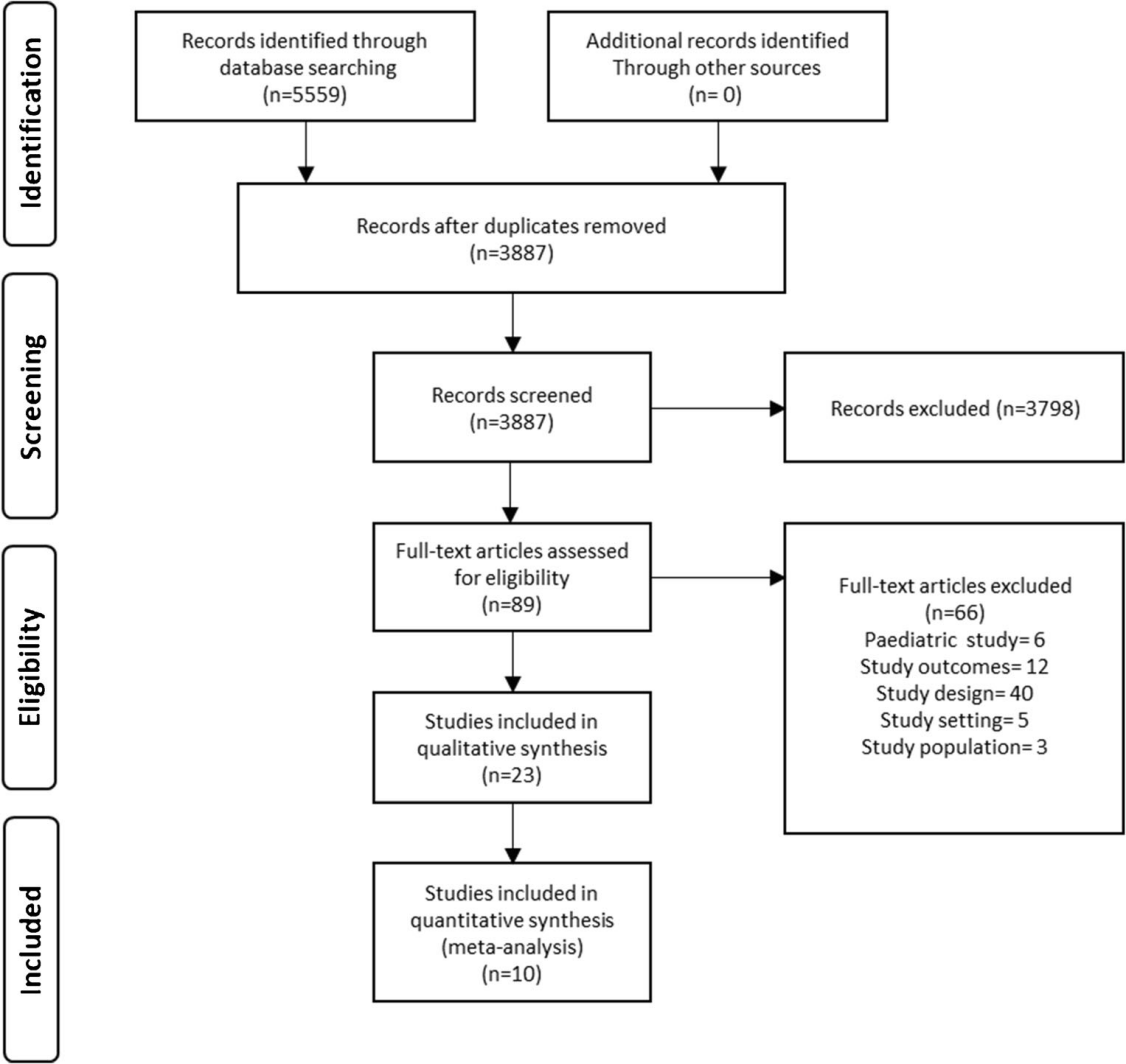
Literature review process

After the removal of duplicates, a total of 3887 papers were identified for screening. Following abstract review, 89 articles were progressed to full-text review. A total of 23 articles were included for qualitative synthesis, of which 10 articles yielded data suitable for meta-analysis [12–21]. These 10 articles examined an aggregate of 3902 sets of radiographs, producing a total of 4709 radiograph interpretation episodes for meta-analysis (see Fig. 2).

The specific anatomical areas examined by articles in the meta-analysis were foot (*n* = 3), ankle (*n* = 4), knee (*n* = 1) and femur (*n* = 2). Two studies examined multiple anatomical locations (see Table 1).

**Table 1:** Table of characteristics for all articles included in meta-analysis

Study author	Anatomical location	Reporters	Sets of radiographs	Gold standard	No. interpretations	Sensitivity	Specificity	Error rate
Utukuri MM, 2000, UK	Foot	2 orthopaedic consultants, 2 orthopaedic trainees, 2 basic surgical trainees and 2 senior emergency medicine staff	50	Known clinical course of patient +/− scans	200 for junior and 200 for senior staff	(one view senior 92.5%, junior 97.5%) (two views senior 100%, junior 97.5%)	(one view senior 86.5%, junior 71.6%) (two views senior 90%, junior 78.5%)	(one view senior 11%, junior 18%) (two views senior 6%, junior 14%)
Vannier MW et al., 1991, USA	Foot	4 MSK radiologists	7	Consensus of plain film and CT	28	95.8%	100.0%	3.6%
York TJ et al., 2020, UK	Ankle	ED doctors ranging in experience from F2 to consultant	2947	Consensus of Orthopaedic Surgeon and MSK radiologist	2947	94.0%	94.8%	5.6%
Singh A.K et al., 1990, UK	Ankle	N/A	114	Consensus of XR and US, if discrepancy then repeat XR performed at 3 weeks	114	85.2%	100.0%	3.5%
Ozturk, P et al., 2018, Turkey	Ankle	1 consultant orthopaedic surgeon	120	CT examination	120	92.8%	100.0%	2.5%
Gray S, 1997, USA	Knee	4 radiology residents	92	Consensus of 3 MSK consultant radiologists with access to follow up imaging	368	(two view 79%, four view 85%)	(two view 87%, four view 92%)	(two view 4.1%, four view 2.7%)
Riaz O, et al. 2016 UK	Femur	2 orthopaedic SpR	289	Intraop findings	578	(two view 54.3%, two views 92.1%)	(one view 89.9%, two view 91.4%)	(one view 16.4%, two view 4.3%)
Lampart A et al., 2019, Switzerland	Femur	Consultant radiologist	70	CT examination	70	82.1%	96.8%	11.4%
Remplik P et al., 2004, Germany	Knee, ankle, foot	2 experienced radiologists	43	Clinical follow-up + review of all imaging	86 (knee 16, ankle 48, foot 22)	58.5% (knee 0%, ankle 66.7%, foot 83.3%)	68.9% (knee 91.4%, ankle 77.7%, foot 100%)	17.4% (knee 31.3%, ankle 25.0%, foot 3.6%)
Ricci et al., 2019, Italy	Extremity fractures	N/A	198	CBCT	198	85.3%	65.5%	20.2%

The studies primarily involved the comparison of plain film radiology with an alternative form of imaging (*n* = 6). Alternatively, inter reader plain film X-ray diagnostic performance was examined (n = 1), or the value of additional X-ray views on diagnostic performance (n = 2), or both (n = 1). The seniority of the studied initial reporters ranged from post-graduate surgical and radiology trainees to senior orthopaedic surgeons, radiologists and emergency physicians.

There was some variation across the ten articles included in the meta-analysis, specifically regarding the definition of a ‘positive’ and ‘negative’ radiographic finding. One article [14] defined positive and negative findings as the presence or absence of any bony or soft tissue pathology. This included soft tissue injury, fractures, dislocations, osteomyelitis and osteoporosis. The other nine articles defined positive and negative finding as the presence or absence of a bony fracture [12, 13, 15–21]. However, two of these nine articles went further and required radiograph interpreters to correctly classify any fracture identified for their findings to be regarded as a ‘true’ positive. Utukuri [12] required interpreters to specify if a calcaneal fracture was intra- or extra-articular. For proximal femur fractures, Riaz O et al. [18] required radiograph interpreters to correctly specify the location and degree of fracture displacement.

### Individual study results

Across all lower limb studies sensitivity ranged from 0.59–0.97, and specificity from 0.66–1.00. Utukuri [12] found the highest sensitivity in initial interpretation, with 0.97 achieved for radiographs of the foot. Ricci [21] found the lowest specificity with only 0.65 achieved for lower limb radiographs (see Table 2).

**Table 2:**
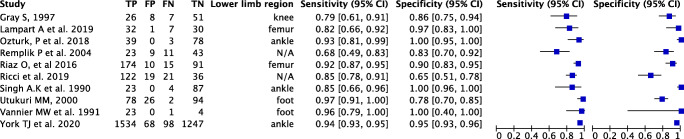
Individual study results forest plot

### Synthesis of results

**Fig. 3:**
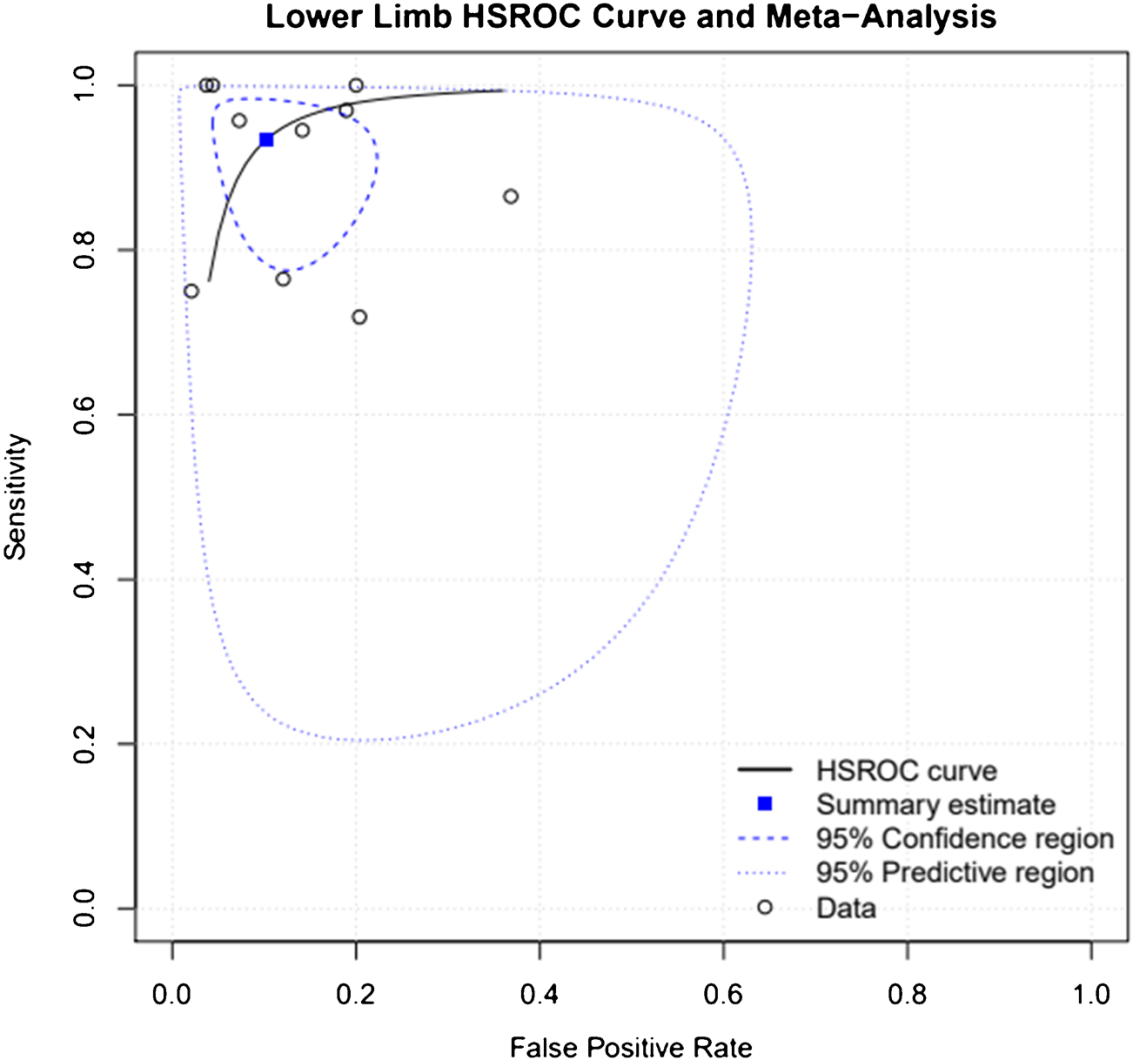
HSROC for all studies

A bivariate model was used to conduct meta-analysis along with a hierarchical summary receiver operating characteristic (HSROC) curve for diagnostic performance across all lower limb plain radiographs (see Fig. 3).

The summary estimate of sensitivity across the included studies was 93.5%, with specificity of 89.7% and a false positive rate of 10.3%. Covariate analysis was also performed to assess specificity and sensitivity by lower limb anatomical subdivision; this was possible for all subdivisions apart from the knee where only a single included study was found (see Table 3).

**Table 3:** Summary estimates

Anatomical region	Sensitivity	Specificity	False positive rate	Diagnostic odds ratio
All studies	0.935	0.897	0.103	125.303
Femur	0.949	0.846	0.154	103
Knee				
Ankle	0.981	0.946	0.054	929.974
Foot	0.949	0.94	0.06	296.168

Summary sensitivity and specificity were both found to be highest for ankle radiographs, 98.1% and 94.6% respectively. Similarly, the initial interpretation of ankle radiographs had the highest diagnostic odds ratio (929.97).

### Risk of bias assessment

All studies included in meta-analysis were analysed using a modified Cochrane risk of bias tool, this qualitative tool assesses study risk of bias on seven separate criteria. One study was considered to be at high risk of bias due to scoring in greater than four categories. Four studies were considered at moderate risk of bias due to scoring in three or more categories or scoring particularly strongly in one of two categories. Five studies scored in two or fewer categories and so were considered to have a low risk of bias (see Table 4).

**Table 4:**
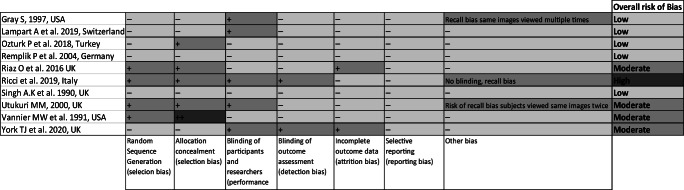
Modified Cochrane ‘Risk of Bias’ assessment tool

### Methodological quality

The methodological quality of the ten articles identified for meta-analysis was assessed using the ‘Minors’ (methodological index for non-randomised studies) tool developed by Slim et al. The range of scores was 13–22 out of a possible 24 points. Articles generally scored highly (average score 16.9).

Nine (90%) of the studies lacked prospective calculations of size, and seven (70%) did not possess an unbiased assessment of their endpoint (see Table 5). Conversely, the studies tended to have minimal losses to follow up (80%) and involved the prospective collection of data (70%).

**Table 5:** Table demonstrating study methodological quality as per MINORS assessment tool

Study author	Clearly stated aim	Inclusion of consecutive patients	Prospective of consecutive patients	Endpoints appropriate to the aim of the study	Unbiased assessment of the study endpoint	Follow-up period appropriate to the aim of the study	Loss to follow-up less than 5%	Prospective calculation of study size	An adequate control group	Contemporary groups	Caseline equivalence groups	Adequate statistical analyses	Total
Gray S, 1997, USA	2	2	2	2	0	2	2	0	1	0	1	2	**16**
Lampart A et al., 2019, Switzerland	2	2	0	2	1	2	2	0	2	2	2	2	**19**
Ozturk, P et al., 2018, Turkey	2	1	2	2	0	2	2	0	2	2	2	2	**19**
Remplik P et al., 2004, Germany	2	2	2	1	0	2	2	0	2	2	0	2	**17**
Riaz O, et al. 2016 UK	2	2	0	2	0	2	0	0	2	2	2	2	**16**
Ricci et al., 2019, Italy	0	1	2	0	0	2	2	0	2	2	2	0	**13**
Singh A.K et al., 1990, UK	0	1	2	1	1	2	2	0	2	2	2	0	**15**
Utukuri MM, 2000, UK	1	0	2	2	0	1	2	0	1	2	2	2	**15**
Vannier MW et al., 1991, USA	2	0	2	2	0	2	2	0	2	2	2	1	**17**
York TK et al., 2020, UK	2	2	0	2	2	2	2	2	2	2	2	2	**22**

## Discussion

### Key findings

This study finds that initial interpreters of lower limb plain radiographs for trauma achieve a relatively high degree of sensitivity (93.5%). It is difficult to quantify the rate at which healthcare systems are justified in accepting the failure to detect findings. Certainly, false negatives are likely to represent the most deleterious of these errors; borne-out by the evidence on litigation for missed fractures both in the UK [6, 22] and abroad [23, 24].

False negatives in the initial interpretation of greater than one in twenty lower limb radiographs, mean that busy accident and emergency or trauma settings are likely to miss substantial numbers of injuries. This appears to support the necessity of safety-netting measures to mitigate the risk of reporting errors. In particular, virtual fracture clinic review [25] and out-of-hours teleradiology services [26] have been widely adopted across the UK and Europe. Alongside these existing methods, the development of novel technologies (such as artificial intelligence algorithms [27]) to supplement interpretation is evidence of a broadly accepted clinical need to improve this reporting.

The summary specificity of reporting was found to be 3.8% lower (89.7%) than sensitivity, suggesting that initial interpreters were less able to identify true negative skeletal radiographs. This finding was commented upon by Utukuri et al. [12] and is also supported by a wider evidence base that shows increasing the seniority of interpreters has a greater benefit to specificity than sensitivity [28, 29]. This implies that some interpretation errors, particularly false negatives, represent a limitation of plain radiographs as a modality and so are not easily preventable. These findings also explain the conclusions of the qualitative synthesis which highlighted the importance of corroborating radiograph interpretation with examination and clinical judgement to prevent fractures being ‘missed’ [7, 30–32].

Of the compared anatomical subdivisions, the diagnostic odds ratio for ankle radiographs was found to be superior, followed by the foot and then the femur. The cause for this is not explored in this study; however, the frequency with which ankle injuries present to emergency and trauma care settings may mean initial interpreters are more practiced in the review of these radiographs. The ankle is both the most commonly injured joint, and also the most frequently operated upon [33]; with the estimated incidence for fractures of the ankle being as high as 187 per 100,000 people per annum [34].

## Limitations

Of the included studies, a generally favourable assessment of risk of bias and methodological quality was made. However, weaknesses were noted due to lack of prospective size calculation and establishing an unbiased endpoint. The extent to which these factors influence results is uncertain; however, sample sizes in a number of studies appear underpowered [12, 19, 20].

During study selection, a number of large sample-size papers were identified but lacked sufficient characterisation of data for inclusion in meta-analysis. Whilst these are a targeted for use in future analysis, they emphasise the importance of reporting diagnostic accuracy along STARD 2015 [35] or similar, relevant guidelines.

## Conclusions

This study suggests that the initial interpretation of plain skeletal radiographs is performed with a relatively high degree of specificity and sensitivity. However, this still represents greater than one in twenty true positives being missed on primary review. The necessity of systems designed to provide safety netting against this are paramount, as are the development of novel means to improve the accuracy of initial interpretation.

Evidence is also found to support statistically significant variation in the accuracy of interpretation across anatomical subdivisions; radiographs of the ankle were shown to have the highest diagnostic odds ratio. The cause of this is uncertain and may reflect inherent difficulties present in certain radiographic views or anatomy, or simply greater interpreter familiarity with some radiographs. Further research is warranted to explore these factors.

## References

[1] Murphy W (1990). Introduction to the history of musculoskeletal radiology. RadioGraphics..

[2] To X-ray or not to X-ray?. Who.int. 2020 [cited 1 October 2020]. Available from: https://www.who.int/news-room/feature-stories/detail/to-x-ray-or-not-to-x-ray-.

[3] Activity H, Accident and Emergency Attendances in England - 2009-2010 E, Accident and Emergency Attendances in England - 2009-2010 E, Accident and Emergency Attendances in England - 2009-2010 E, Accident and Emergency Attendances in England - 2009-2010 E, Accident and Emergency Attendances in England - 2009-2010 E et al. Accident and Emergency Attendances in England - 2009-2010, Experimental statistics - NHS Digital [Internet]. NHS Digital. 2020 [cited 26 August 2020]. Available from: https://digital.nhs.uk/data-and-information/publications/statistical/hospital-accident%2D%2Demergency-activity/2009-2010.

[4] de Lacey G (1979). Number of casualty attenders referred for X-ray examination. Br J Radiol.

[5] Guly H (2001). Diagnostic errors in an accident and emergency department. Emerg Med J.

[6] Halpin S (2009). Medico-legal claims against English radiologists: 1995–2006. Br J Radiol.

[7] Petinaux B, Bhat R, Boniface K, Aristizabal J (2011). Accuracy of radiographic readings in the emergency department. Am J Emerg Med.

[8] Kranz R, Cosson P (2015). Anatomical and/or pathological predictors for the “incorrect” classification of red dot markers on wrist radiographs taken following trauma. Br J Radiol.

[9] Landis J, Koch G (1977). The measurement of observer agreement for categorical data. Biometrics..

[10] Slim K, Nini E, Forestier D, Kwiatkowski F, Panis Y, Chipponi J (2003). Methodological index for non-randomized studies (MINORS): development and validation of a new instrument. ANZ J Surg.

[11] Sterne J, Savović J, Page M, Elbers R, Blencowe N, Boutron I et al. RoB 2: a revised tool for assessing risk of bias in randomised trials. BMJ. 2019;l4898. 10.1136/bmj.l4898 31462531

[12] Utukuri M, Knowles D, Smith K, Barrie J, Gavan D (2000). The value of the axial view in assessing calcaneal fractures. Injury..

[13] Vannier M, Hildebolt C, Gilula L, Pilgram T, Mann F, et al. Calcaneal and pelvic fractures: diagnostic evaluation by three-dimensional computed tomography scans. J Digit Imaging. 4(3):143–52. 10.1007/BF03168159 1911972

[14] York T, Jenkins P, Ireland A (2019). Reporting discrepancy resolved by findings and time in 2947 emergency department ankle X-rays. Skelet Radiol.

[15] Singh A, Malpass T, Walker G (1990). Ultrasonic assessment of injuries to the lateral complex of the ankle. Emerg Med J.

[16] Ozturk P, Aksay E, Oray N, Bayram B, Basci O, Tokgoz D (2018). Emergency physician accuracy using ultrasonography to diagnose lateral malleolar fracture. Am J Emerg Med.

[17] Gray S, Kaplan P, Dussault R, Omary R, Campbell S, Chrisman H (1997). Acute knee trauma: how many plain film views are necessary for the initial examination?. Skelet Radiol.

[18] Riaz O, Nisar S, Arshad R, Vanker R (2016). Lateral X-ray for proximal femoral fractures – is it really necessary?. Surgeon.

[19] Lampart A, Arnold I, Mäder N, Niedermeier S, Escher A, Stahl R (2019). Prevalence of fractures and diagnostic accuracy of emergency X-ray in older adults sustaining a low-energy fall: a retrospective study. J Clin Med.

[20] Merl T, Roemer F, Bohndorf K, Remplik P, Stabler A (2004). Diagnosis of acute fractures of the extremities: comparison of low-field MRI and conventional radiography. Eur Radiol.

[21] Ricci P, Boldini M, Bonfante E, Sambugaro E, Vecchini E, Schenal G (2019). Cone-beam computed tomography compared to X-ray in diagnosis of extremities bone fractures: a study of 198 cases. Eur J Radiol Open.

[22] Hulbert D, Riddle W, Longstaff P, Belstead J, Beckett M (1996). An audit of litigation costs in four accident and emergency departments. Emerg Med J.

[23] Deakin A, Schultz T, Hansen K, Crock C (2014). Diagnostic error: missed fractures in emergency medicine. Emerg Med Australasia.

[24] Ahmed S, DeFroda S, Naqvi S, Eltorai A, Hartnett D, Ruddell J (2019). Malpractice litigation following traumatic fracture. J Bone Joint Surg.

[25] Logishetty K (2017). Adopting and sustaining a virtual fracture clinic model in the district hospital setting – a quality improvement approach. BMJ Qual Improv Rep.

[26] Dixon A, FitzGerald R (2008). Outsourcing and teleradiology: potential benefits, risks and solutions from a UK/European perspective. J Am Coll Radiol.

[27] Jones R, Sharma A, Hotchkiss R, Sperling J, Hamburger J, Ledig C et al. Assessment of a deep-learning system for fracture detection in musculoskeletal radiographs. npj Dig Med. 2020;3(1). 10.1038/s41746-020-00352-w PMC7599208 33145440

[28] Brealey S, Scally A, Hahn S, Thomas N, Godfrey C, Coomarasamy A (2005). Accuracy of radiographer plain radiograph reporting in clinical practice: a meta-analysis. Clin Radiol.

[29] Loughran C (1994). Reporting of fracture radiographs by radiographers: the impact of a training programme. Br J Radiol.

[30] Clark T, Janzen D, Ho K, Grunfeld A, Connell D (1995). Detection of radiographically occult ankle fractures following acute trauma: positive predictive value of an ankle effusion. Am J Roentgenol.

[31] Comat G, Barbier O, Ollat D (2014). The posterior malleolar fracture: a parachute injury not to be overlooked. Orthop Traumatol Surg Res.

[32] Lewis SL, Rees JI, Thomas GV, LA TW (1991). Pitfalls of bone scintigraphy in suspected hip fractures. Br J Radiol.

[33] Bauer M, Bengnér U, Johnell O, Redlund-Johnell I (1987). Supination-eversion fractures of the ankle joint: changes in incidence over 30 years. Foot Ankle.

[34] Daly P, Fitzgerald R, Melton L, Llstrup D (1987). Epidemiology of ankle fractures in Rochester, Minnesota. Acta Orthop Scand.

[35] Cohen J, Korevaar D, Altman D, Bruns D, Gatsonis C, Hooft L (2016). STARD 2015 guidelines for reporting diagnostic accuracy studies: explanation and elaboration. BMJ Open.

